# Defining the cost of the Egyptian lymphatic filariasis elimination programme

**DOI:** 10.1186/1475-2883-4-7

**Published:** 2005-08-02

**Authors:** Reda MR Ramzy, Ann S Goldman, Hussein A Kamal

**Affiliations:** 1 Research & Training Center on Vectors of Diseases, Ain Shams University, Cairo, Egypt; 2 Department of Epidemiology and Biostatistics, The George Washington School of Public Health and Health Services, Washington, DC, USA; consultant to the Emory Lymphatic Filariasis Support Center, Emory University, Atlanta, USA; 3 Ministry of Health & Population, Cairo, Egypt

## Abstract

**Background:**

Lymphatic filariasis (LF) is targeted for global elimination. LF elimination programmes in different countries, including Egypt, are supported financially by national and international agencies. The national programme in Egypt is based on mass drug administration (MDA) of an annual dose of a combination of 2 drugs (DEC and albendazole) to all endemic villages. This study aimed primarily to estimate the Total and Government costs of two rounds of MDA conducted in Egypt in 2000 and 2001, the average cost per person treated, and the cost share of the different programme partners.

**Methods:**

The Total costs reflect the overall annual costs of the MDA programme, and we defined Government costs as those expenditures made by the Egyptian government to develop, implement and sustain the MDA programmes. We used a generic protocol developed in coordination with the Emory Lymphatic Filariasis Support Center. Our study was concerned with all costs to the government, donors and other implementing parties. Cost data were retrospectively gathered from local, regional and national Ministry of Health and Population records. The total estimates for each governorate were based on data from a representative district for the governorate; these were combined with national programme data for a national estimate.

**Results:**

The overall Total and Government costs for treating approximately 1,795,553 individuals living in all endemic villages in the year 2000 were US $3,181,000 and US $2,412,000, respectively. In 2001, the number of persons treated increased (29%) and the Total costs were US $3,109,000 while Government costs were US $2,331,000. In 2000, the average Total and Government costs per treated subject were US $1.77 and $1.34, respectively, however, these costs decreased to US $1.34 and $1.00, respectively in 2001. The coverage rate was 86.0% in 2000 and it increased to 88.0% in 2001.

**Conclusion:**

The Egyptian government provided 75.8% of all resources, as reflected in the Total cost estimates, and international agencies contributed the rest. Such data highlight both the commitment of the Egyptian government and the significance of the contributions of international bodies toward the LF elimination programme.

## Background

Lymphatic filariasis (LF), also known as elephantiasis is caused by infection with the threadlike nematode *Wuchereria bancrofti *and transmitted by Culex pipiens mosquitoes; it is known to be endemic in rural areas of Egypt. Administratively, Egypt consists of 26 governorates with a population currently estimated at 68 million people, more than 60% residing in the densely populated governorates of the Nile Delta. The disease has a focal distribution, and it is estimated that currently over 2.5 million people are at risk of acquiring *W. bancrofti *infection. LF is considered one of the most important vector borne diseases in Egypt, posing a major public health problem in 6 governorates in the Nile Delta and the governorates of Giza and Asiut in Upper Egypt.

In Egypt the Malaria, Filariasis and Leishmaniasis Control Department of the Ministry of Health and Population (MOHP) has worked since the 1970s to map and decrease LF prevalence by focalized treatment initiatives to reduce transmission. Selective treatment of microfilaremic subjects with a 12-day regimen of diethylcarbamazine (DEC; 6 mg/kg/day) was recommended. In 1996, the MOHP changed its anti-LF strategy to selective treatment with single-dose DEC (6 mg/kg) as it was shown to be equally effective[1]. In 1997, the World Health Assembly passed a resolution (50.97) calling for "the elimination of lymphatic filariasis as a public health problem." Consequently, the World Health Organization (WHO) developed a new strategy and initiated a global programme for the elimination of lymphatic filariasis as a public health problem by the year 2020[1].

Egypt was among the first countries to join the WHO global efforts. A national programme to eliminate LF as a public health problem was initiated in the year 2000. In the years of the focalized control campaign Egypt had made substantial progress in decreasing microfilaria prevalence. Thus, upon joining the global elimination effort, the programme sought specifically to decrease the microfilaria prevalence rates to less than 0.1%. The programme, which is based on mass drug administration (MDA) of an annual dose of DEC (6 mg/kg) in combination with albendazole (400 mg), aims to achieve an MDA coverage rate of about 80% of the total population of the target implementation units (IU). The village was chosen as the IU, and all villages with microfilaria or an antigen prevalence rate of 1% or more were included in the LF elimination programme. The elimination programme, based within the MOHP, is financially supported also by other partners within the government, including the Ministries of Agriculture and Information, and by international agencies such as WHO (Geneva) and its Eastern Mediterranean Regional Office (EMRO, Cairo), as well as private donors like GlaxoSmithKline (GSK). The Research and Training Center on Vectors and Diseases (RTC) at Ain Shams University (ASU) has been collaborating with the programme on consultation, training, and surveillance activities.

The LF elimination programme is managed by the Malaria, Filariasis and Leishmaniasis Control Department Headquarters (PHQ) office, supported by a strategic steering committee. The PHQ office has overall responsibility for the administration of all MDA activities at the central level, as well as oversight of activities at the peripheral (governorate, district and village) levels. District level filariasis units report data on LF mapping and surveillance following MDA rounds to the PHQ office. The Ministry of Agriculture through its guidance units, also at the district level, participated in local social mobilization activities. The MOHP infrastructure contains a well-developed network of rural health centers (RHC) to provide health services at the village level. RHC physicians, nurses and health workers in target villages carry out drug distribution and other MDA related activities. Support for the public education and mobilization campaigns came from the Ministry of Information that broadcasts materials in various media at the national level. The Ministry of Religion participates on the steering committee. This participation does not entail any additional financial support. In addition, the steering committee liaises with local community leaders to enlist their cooperation in encouraging the general public to participate in the MDA campaign. MDA activities included:

1) Mapping of LF endemic villages based on previous data and conducting surveys using a rapid format card test for villages with uncertain LF situation or outdated data. The population census of the target villages was then updated.

2) Training sessions for physicians and nurses working in the RHC of the target villages.

3) Social mobilization activities that consisted of meetings with local village leaders, distribution of pamphlets and posters, short advertising programmes for TV and radio to create population awareness and facilitate community participation.

4) House to house drug distribution, where RHC distributors observed pill consumption.

5) Treatment of adverse reactions to the drugs.

6) Surveillance of residual infection by assessment of microfilaremia prevalence rates in selected sentinel villages.

7) Administration of the different programme activities at different levels.

Supported by a grant from the Bill & Melinda Gates Foundation, the Emory Lymphatic Filariasis Support Center (Emory LFSC) is coordinating a multi-center cost analysis study of LF elimination programmes, the first of its kind in the field of LF. In 2002, the RTC at ASU in collaboration with the Egyptian MOHP joined the initiative to carry out a study of two MDA rounds implemented in 2000 and 2001 as part of the LF elimination programme in Egypt. The primary objectives were to estimate the Total cost and Government cost of the LF programme in 2000 and 2001, estimate the average cost per person treated, identify the programme elements with the greatest contribution to overall costs, and estimate the cost share of the different programme partners.

## Methods

To achieve the above-mentioned objectives, a generic protocol was developed and field-tested as part of the nine-country Emory LFSC study [2]. The protocol was designed to provide methodological guidance to investigators and create a systematic approach for country-specific data collection and analysis so that cost estimates would be comparable across a variety of settings with different conditions. The study was concerned with all costs to the government, donors and other implementing parties. The medication for the MDA was distributed to the population free of charge, as was any medication to treat adverse reactions. Costs to persons receiving treatment were not investigated. The estimates for the MDA costs were obtained through retrospective data collection.

Countries participating in the nine-country Emory LFSC study went through a process of cost identification and agreed to cost the following functions: training, mapping, mobilization and education, drug distribution, adverse reaction monitoring and treatment, surveillance/laboratory, and administration (See Table 1). Two other categories were added, "LF-non MDA costs" and "Other, non-LF, non-MDA costs", to help distinguish the resources for the MDA activities from other LF and MOHP activities. These two categories of costs were ultimately excluded from the MDA programme cost calculations.

**Table 1:** MDA Activities and other cost categories

Activity	Definition
*Training*	Instruction of MOH personnel to carry out the administrative and functional activities of the MDA and instruction of volunteers to develop skills required for the MDA.
*Mapping*	Testing to establish microfilaria prevalence in communities.
*Mobilization and Education*	Media campaigns and community activities to increase MDA participation.
*Drug Distribution*	Logistic aspects of management of the drugs as well as administration of the drugs to the population.
*Adverse Reaction Monitoring*	Observation and treatment of persons suffering adverse reactions due to the MDA.
*Surveillance and Laboratory*	Tracking of community members in MDA area, laboratory work for testing, case identification, etc.
*Administration*	Supervisory work and paperwork to support the MDA.
*LF Non-MDA Costs*	Costs related to LF, but not the MDA (excluded from cost calculations).
*Other Non-LF, Non-MDA Costs*	MOH costs not at all related to the MDA or to LF (excluded from cost calculations).

The costing was divided into the input categories of medications and laboratory supplies (including: medications for the MDA and adverse reaction treatment, as well as laboratory supplies), personnel, transport, general supplies, and recurrent and capital costs for facilities and equipment. Capital costs refer to expenditures for inputs purchased for use of one or more years. Capital goods were annualized using a 3% discount rate. These inputs can be vehicles, facilities or equipment. The calculations took into account years of useful life of the equipment, according to Ministry of Finance schedules. When information about capital goods was not available, the value of renting the item was used and considered a recurrent cost. Recurrent costs are those incurred for goods and services purchased and used up on a regular basis, such as personnel time, most supplies, fuel, etc. Cost information was gathered for the programme inputs from local, regional and national MOHP records. This information and records of recurrent costs provided information on the percentage of time and resources devoted to the programme. In addition, programme managers were interviewed to estimate the percentage of time personnel devoted to the LF programme.

Drug cost information was provided to the Emory LFSC and the participating countries by GSK (donated albendazole), and the WHO (purchased DEC). Data on MDA coverage rates to aid in the calculation of cost per person treated and documentation of personnel salaries and special LF incentives came from MOHP district and national files. The cost of supplies for the MDA, such as drug tablets, ICT cards, adverse reaction medications, etc. was collected at the national level. The PHQ office keeps records of the number of drug tablets and ICT cards distributed per village. The costs of training and social mobilization initiatives were also estimated from PHQ and Ministry of Information records. The Ministry of Agriculture provided information on resources it devoted to the MDA. Estimates for transportation, recurrent and capital costs were based upon interviews with local personnel at the district level.

Data were collected from one district in each governorate and combined with national level cost information about the number of villages, RHCs, categories and numbers of personnel in each of these, and aggregated LF incentives for each district. The districts chosen for the analysis were chosen at random unless the governorate contained only one district. In one instance, the governorate contained two districts and the district chosen was the largest. These data were used to calculate approximations for other districts. Thus, the total estimates for each governorate were based on data from what was considered a representative district for the governorate combined with detailed information obtained from the national government about categories and numbers of personnel, their salaries and any incentives that were offered.

The activities of some MDA partners like Ain Shams University's RTC, which participated in personnel training and monitoring and evaluation of the MDA, are considered as part of the MDA but not separately attributed to these partners since they were funded with central MOHP monies. In the case of other partners like the Ministry of Religion and community leaders, obtaining documentation and monetary quantification of their contributions in assisting in garnering public support for the MDA was difficult to do.

This cost analysis sought to estimate a Total programme cost, accounting for all resources used in carrying out the programme, including donations of supplies, equipment, time, etc. This approach is useful in evaluating the allocation of programme resources and their opportunity costs, i.e., whether these resources could be used more productively elsewhere. Government costs are helpful to programme managers in looking at actual programme expenditures and assessing affordability. We defined Government costs as the costs of all inputs paid for by the Ministry of Health, excluding any direct donations to the programme. Any donations such as albendazole were considered part of Total costs, as were any donations external to the government such as the community or local or foreign donors. Because the capital costs were annualized, the cash expenditures described reflect those incurred in one year for the project.

All costs were prepared in Egyptian pounds (LE), and the cost of direct supplies donated or provided by different international agencies were estimated in US dollars (US $) and converted to equivalent LE amounts. For this paper, costs are presented in US dollars. The exchange rates used for costs estimates in 2000 and 2001 were 3.47 and 3.88 LE for US $1.00, respectively. No adjustment was made for inflation as inflation was negligible in the United States during the years covered by the analysis. Conversion to U.S. dollars accounted for Egyptian inflation.

## Results

### Mass Drug Administration Coverage Rate for Calculation of Total and Government Costs

In 2000, MDA was implemented in 161 villages in 7 governorates. Coverage rates (treated at-risk population divided by total at-risk population) calculated using the data collected by RHC distributors ranged among the different governorates from 78.6 to 89.9%. The overall MDA coverage rate was 86.0% (Table 2). In 2001, the MDA was carried out in 178 villages in eight governorates with coverage between 85.7 and 95.9%. That year, the estimated overall MDA coverage rate increased significantly to 88.0% (*X*^2 ^= 4007, p < 0.0001) (Table 2).

**Table 2:** MDA coverage rates for 2000 and 2001

**Governorate**	**2000**				**2001**				**% increase in treated population**
		
	**No. of districts**	**No. of Treated villages**	*Treated population	**MDA coverage (%)**	**No. of districts**	**No. of Treated villages**	**Treated population**	**MDA coverage (%)**	
**Qalyubia**	7	68	818,488	82.9	7	68	890,967	85.7	9
**Menofia**	4	24	190,024	88.8	4	25	284,786	88.4	50
**Sharkia**	6	32	272,722	84.4	6	32	324,762	89.1	19
**K. El Sheikh**	1	1	18,200	78.6	1	2	32,972	95.9	81
**Dakahlia**	1	24	192,063	89.9	1	36	426,588	95.4	122
**Gharbia**	2	2	51,782	87.7	2	2	57,638	90.9	11
**Giza**	4	10	216,274	80.8	4	11	237,679	85.7	10
**Assiut**					2	2	65,210	87.6	
**Total**	25	161	1,795,553	86.0	27	178	2,320,602	88.0	29

*Treated population is defined as the number of persons who ingested the drugs, based on family forms filled by drug distributors.

### Total and Government costs

**Figure 1:**
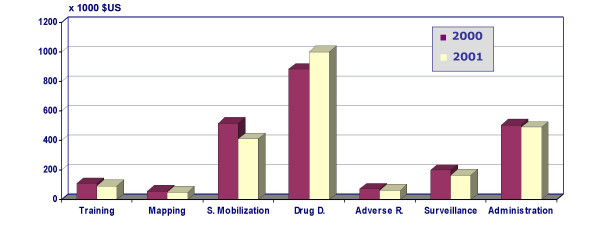
Government costs of the different programme activities during the MDA in 2000 and 2001.

The overall Total and Government costs for treating approximately 1,795,553 individuals living in all endemic villages covered by the MDA in 2000 were US $3,181,000 and US $2,412,000, respectively. In 2001, the number of persons treated increased (29%) to approximately 2,320,602, and the Total and Government costs were US $3,109,000 and US $2,331,000, respectively. In both years, for the Total costs the largest proportion of this amount was spent for drug distribution (40.9% and 45.9% for 2000 and 2001, respectively), followed by programme administration (20.1% and 20.8% for 2000 and 2001, respectively). A similar pattern of expenditure was also observed for Government costs. Figure 1 shows Government costs of the two MDA rounds (2000 and 2001) as presented by programme activities.

**Figure 2:**
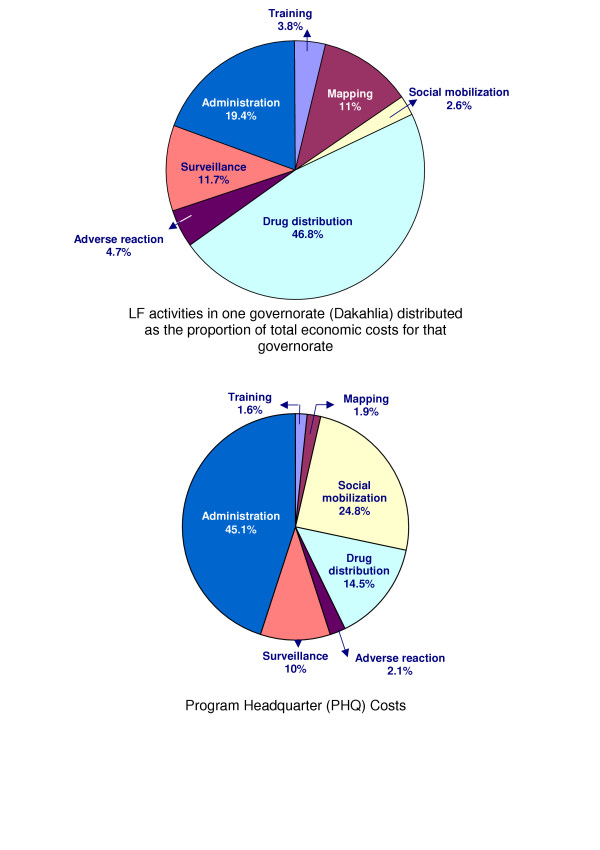
MDA Total cost share % for programme activities as presented for the Programme Head Quarter (PHQ) and a representative governorate in 2000.

Generally, there was a high degree of uniformity among expenditures in all governorates for the different programme activities. Note that the costs of mapping, treatment of drug adverse reactions and social mobilization at the governorate level were relatively low (Figure 2). The cost of central social mobilization (mainly national TV and radio broadcasting) was included in PHQ expenses and accounted for about 24.8% of the central programme expenses (Figure 2).

**Figure 3:**
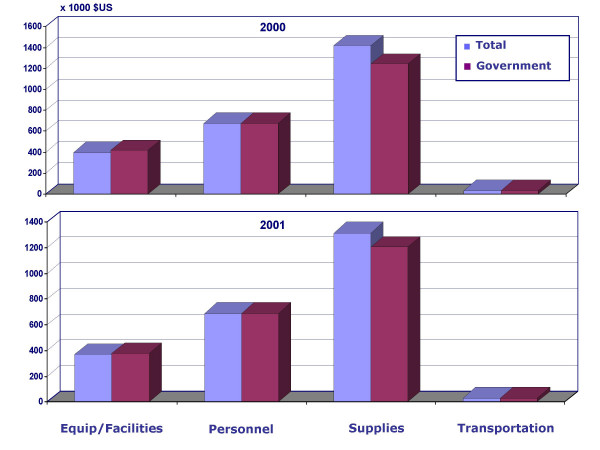
2000 and 2001 MDA Total and Government costs distributed by programme inputs.

The distribution of Total and Government costs by programme items including personnel salaries and incentives, equipment and programme facilities, supplies and transportation showed similar patterns (Figure 3). For Total costs, the highest expenditure was in the category of supplies (56.5% and 52.7% for 2000 and 2001, respectively) followed by personnel (27.0% and 30.1% for 2000 and 2001, respectively). The lowest programme expenditure was for transportation since almost all villages were served by local RHCs. Thus for the most part teams distributing drugs worked in the same villages and did not move very far. The majority of transportation that took place in the MDA programme was for sending drugs from MOHP central storage to RHCs and for supervision by the district director.

### Comparison of MDA costs for governorates covered by MDA in the 2 years

There was a wide range (9%-122%) in the increases of persons treated in the governorates with at risk populations during the 2001 MDA compared to 2000. The greatest increase was in Dakahlia (122%) followed by the governorates of Kafr El Sheikh (81%) and Menofia (50%). The overall increase in treated population in 2001 was 29% (Table 2). In terms of cost, the overall programme Total and Government costs as expressed in Egyptian pounds increased 9.6% and 7.8%, respectively. However, these costs as expressed in US dollars decreased 2% and 3% due to the re-evaluation of the pound against the dollar (Table 3). The overall cost of personnel increased 13.2%, particularly in Dakahlia (73.6%), Kafr El Sheikh (29.6%) and Menofia (14.7%) governorates (data not shown). Nonetheless, the overall Total and Government costs for supplies; equipment and programme facilities; and transportation did not change significantly (<10%). However, in 2001 a significant increase in supplies caused Government costs to rise in Dakahlia (77.2%), Kafr El Sheikh (36.8%) and Menofia (37.3%) governorates, whereas the costs for equipment and programme facilities, and transportation increased only in Dakahlia governorate (43.7% and 38.6%, respectively; data not shown).

**Table 3:** Comparison Between Government, Total Costs and Cost per Person Treated in 2000 and 2001

	**Total Government and Total Costs and Cost per Person Treated**							
	
	**2000**				**2001**			
	
	**Costs (US$)**		**Cost per person treated (US$)**		**Costs (US$)**		**Cost per person treated (US$)**	
	
**Programme Level**	**Government Cost**	**Total Cost**	**Government Cost**	**Total Cost**	**Government Cost**	**Total Cost**	**Government Cost**	**Total Cost**
***Governorate level***								
**Qalyubia**	696,000	949,000	0.85	1.16	653,000	913,000	0.73	1.03
**Menofia**	264,000	320,000	1.39	1.68	275,000	358,000	0.97	1.26
**Sharkia**	325,000	405,000	1.19	1.48	308,000	403,000	0.95	1.24
**K. Sheikh**	30,000	36,000	1.65	1.96	32,000	42,000	0.98	1.28
**Dakahlia**	220,000	281,000	1.14	1.46	322,000	452,000	0.75	1.06
**Gharbia**	59,000	74,000	1.14	1.44	56,000	73,000	0.97	1.27
**Giza**	181,000	249,000	0.84	1.15	172,000	241,000	0.7	1.02
***National level***								
**PHQ**	637,000	867,000	0.35	0.48	513,000	627,000	0.22	0.27
**TOTAL**	2,412,000	3,181,000	1.34	1.77	2,331,000	3,109,000	1.00	1.34

*Assiut is not included in Table 3 as MDA commenced in 2001.

### Average cost per treated subject

In 2000, the average Total and Government costs per treated subject were US $1.77 and US $1.34, respectively. In 2001, however, these costs decreased to US $1.34 and US $1.00, respectively. The ranges for average Total costs per treated person among the different governorates for 2000 and 2001 were US $1.15–1.96 and US $1.02–1.28, respectively (Table 3). A similar pattern was observed for the Government costs per person treated among the different governorates (Table 3).

### Contribution of different partners to the elimination programme

As stated above the Total costs reflect the overall annual costs of the MDA programme. We defined as Government costs those expenditures made by the Egyptian government to develop and sustain the MDA programmes. All donations, including community donations were excluded from the Government cost calculations. In Egypt the overall Total costs were US $3,181,000 in 2000 and US $3,109,000 in 2001. After excluding donations for medication (DEC and albendazole), ICT cards and the cost of developing TV video advertisements, Government costs were US $2,412,000 in 2000 and US $2,331,000 in 2001.

**Figure 4:**
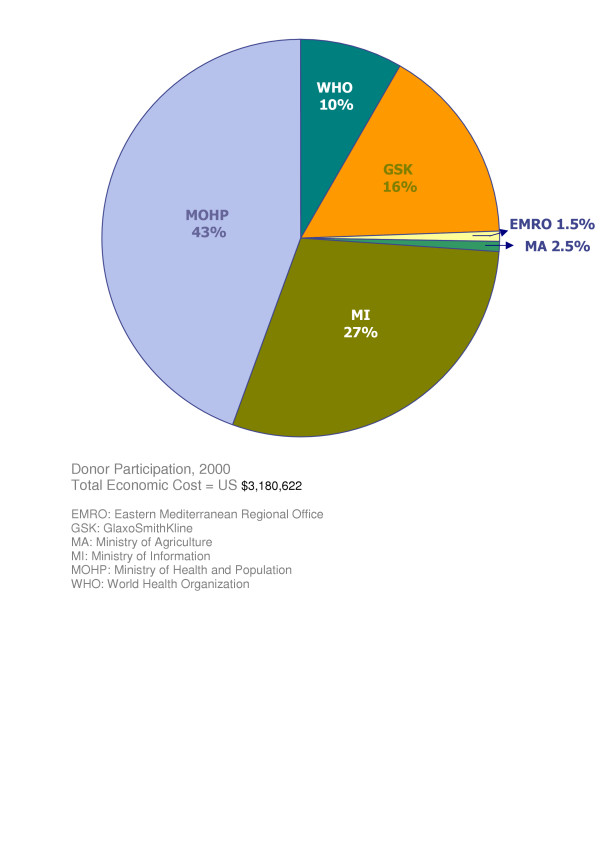
Contributions of national and international partners to the Egyptian LF elimination programme in 2000.

Figure 4 shows the relative contribution of the different programme partners in 2000. WHO provided DEC and the cost of producing TV video advertisements which accounted for 6.7% of the total programme expenses; GSK donated albendazole, 16%; EMRO provided ICT cards, 1.5%; the Ministry of Agriculture provided 2.5% in kind through the use of agriculture guidance units, the Ministry of Information provided the cost of national TV broadcasting, 27%; and the remaining 46.3% was from the MOHP resources. Overall, the government of Egypt contributed 75.8% of the Total costs. A similar pattern of contributions by the same partners was observed for 2001 (data are not shown).

### Contribution of MOHP to MDA cost in relation to the overall MOHP expenditures in the studied districts

Table 4 summarizes the contribution of the MOHP to the cost of MDA (2000) in relation to the MOHP total expenditure for endemic diseases in the studied districts as presented by governorate. In general, there was a close range from 9% for Qalyubia governorate to 12% for K. Sheikh governorate. The overall MOHP contribution to the cost of this MDA round accounted for 10% of the total MOHP expenditure for endemic diseases. Data for 2001 (not shown) were similar to these obtained for 2000.

**Table 4:** MOHP contribution to cost of MDA (2000) in relation to MOHP total endemic diseases expenditure

**Region**	MOHP* expenditure	**Cost of MDA (MOHP)**	**%**
**Qalyubia**	$3,868,000	$357,000	9
**Menofia**	$1,252,000	$132,000	11
**Sharkia**	$1,514,000	$162,000	11
**K. Sheikh**	$126,000	$15,000	12
**Dakahlia**	$1,057,000	$111,000	11
**Gharbia**	$292,000	$30,000	10
**Giza**	$963,000	$93,000	10
**Total**	$9,072,000	$900,000	10

*Estimated MOHP expenditure, in US dollars, on endemic diseases in target district presented by governorate.

## Discussion

The present work is part of a multi-center study using a generic protocol to analyze the Total and Government costs of LF elimination programmes in different settings with diverse field and programmematic conditions. This study sought to estimate and analyze the Total costs of Egypt's LF programme for the first two MDA rounds implemented in 2000 and 2001. Data were retrospectively collected for each year separately. In analyzing the data, we distinguished between Total and Government costs that provide different but complementary information. We defined as Government costs those expenditures made by the government of Egypt to develop, implement and sustain the MDA programmes. All donations, including community donations were excluded from the Government cost calculations.

Analysis of Total costs, restricted to MDA activities, revealed that the average cost per treated subject decreased in 2001 (US $1.34) from 2000 (US $1.77). This is largely the result of economies of scale, in that the programme depended on the MOHP's well articulated health system infrastructure and use of the same capital items (RHC buildings, laboratory equipment, etc) to treat and serve a larger number of persons. In support of this conclusion it should be noted that although the number of treated subjects increased (29%) in 2001, the average costs per treated person decreased (28.5%), and the cost of personnel and other inputs increased by only 13%. Currency exchange rate changes tended to magnify the observed decrease in cost. But even normalizing the 2001 exchange to equal that in 2000, yielded a cost per treated subject of $1.50 that is still less than the 2000 figure of $1.77. The exchange rate in 2001 (3.88 LE for US $1.00) was higher than in 2000 (3.47 LE for US 1.00).

The average per-treatment Total cost for implementing the first annual MDA round (US $1.77) was comparable to that reported for southern India in 2002 (US $1.49)[3], based on an annual DEC-ivermectin regimen, and higher than that calculated in Tanzania in 1996 (US $0.70)[4], based on a semi-annual DEC mass treatment. Government costs for the two years of MDA were US $1.34 in 2000 and US $1.00 in 2001. Nonetheless, this information is intended, not really as a comparison, but more to help to contextualize the results of the Egypt study. A number of elements could partially account for the cost difference among programmes in Egypt, India and Tanzania, including, the added cost of either albendazole or ivermectin and the fact that different measures may have been used in the analyses, and different time periods analyzed. The Emory LFSC multi-country cost analysis project, which included the costing of the Egypt MDA programme in 2001 and 2002, reported here, was developed to facilitate comparability between country programmes by establishing definitions for inputs and activity categories.

The MDA implementation units (IUs) consisted primarily of rural agricultural villages with comparable configurations and structures of health systems but different population sizes, and a few semi-urbanized towns. The similarity among IUs may explain the observed high degree of uniformity in the structure of costs for the different programme activities among endemic governorates. Indeed, overall governorate Total and Government costs scale up proportionally to the size of treated populations (Table 3).

We noted that the costs of mapping, treatment of drug adverse reactions and social mobilization at the peripheral levels (village, district and governorate) were relatively low (Figure 2). No doubt that the low mapping cost was due to the fact that most LF endemic villages were already known to MOHP from previous data, and the use of the card test was restricted to mapping the relatively few villages with an uncertain LF situation or outdated data.

Adverse events after the first MDA were uncommon and mostly of mild to moderate severity, the most frequently observed being fever, headache, and myalgia. These symptoms, believed to be mainly due to dying worms, usually resolved within two to three days[5]. Adverse reactions following the second round of MDA were greatly reduced compared to those observed following the first round. These observations would greatly explain the low cost of the treatment of drug adverse reactions.

The two MDA rounds studied, implemented in 2000 and 2001, were considered very successful, as the overall MDA coverage rates reached 86.0% and 88.0%, respectively (Table 2). While high treatment coverage is not always found in MDA programmes elsewhere [6-8] in the current programme different social mobilization approaches were implemented at different central and peripheral levels to secure high MDA coverage. Estimated costs of social mobilization at the village and district levels were quite low (about 2–3% of the total governorate expenditure) but is worth noting that the cost of one critical social mobilization activity, involvement of religious leaders at the village level, could not be quantified in the current study. A Moslem leader, for instance, would dedicate a few minutes of his speech (usually about 30 minutes long) at Friday prayers on two to three occasions to emphasize the importance of the MDA programme and to encourage audiences to take the treatment; on the other hand, the estimated central cost for social mobilization (national TV and radio broadcastings) was quantifiable and quite high (Figure 4), accounting for about 27% of the total programme expenditure.

The number of persons treated increased 29% in 2001 over 2000. This was the result of normal population growth (estimated country growth rate 2.1%) in all villages, of adding new implementation units particularly in Dakahlia governorate (12 villages), and of including small hamlets (satellites to villages) as in the case of Menofia governorate. In Kafr El Sheikh governorate, only one village was added in 2001 but this resulted in a doubling of the number of persons treated (Table 2). When Total and Government costs were compared between the two years, the costs of personnel increased 13.2%. For the most part, this reflects participation of more manpower (physicians, nurses and other health workers) particularly in Dakahlia and Kafr El Sheikh governorates or increased person work days to cover the newly added satellite villages as in the governorate of Menofia. Generally, however, the Total and Government costs for supplies did not change significantly in the governorates with the exception of Dakahlia, Kafr El Sheikh and Menofia. Costs of equipment and programme facilities increased in the governorate of Dakahlia only, as a result of using more capital items and RHC facilities in 2001. Similarly, the cost of transportation increased in Dakahlia only, as a consequence of the costs of supervising the MDA in more implementation units.

We also analyzed the MOHP contribution to the Government cost of MDA (2000) in relation to the total MOHP expenditure for endemic diseases in the studied districts and grouped them by governorate. The analysis showed that the cost of MDA represents a relatively small cost burden (10%) on the MOHP budget for endemic diseases.

The LF elimination programme was financially supported by several national and international partners. Our data (Figure 4) indicated that national agencies contributed about 75.8% (cash or in kind) of the Total costs highlighting the commitment of the Egyptian government toward the LF elimination programme. This does not minimize the significance of the contributions of international bodies. GSK provided all albendazole tablets free of charge, WHO (Geneva) purchased DEC tablets, EMRO supplied card tests through a contribution from the Arab Fund for Economic and Social Development.

Based on MOHP data before the initiation of the LF elimination programme, about 314 villages had a history of LF endemicity [9]. Currently, 178 villages, with >1% LF prevalence, are included in the national elimination programme and covered by multiple MDA rounds. Many of the remaining villages are believed to have infection rates <1%, based on data acquired before 1990. However, mapping activities undertaken in certain governorates (e.g. Dakahlia governorate) in 2000 and 2001 revealed that 12 villages have >1% LF prevalence rates and were therefore added to the MDA programme. In this context, the MOHP is now required to map the remaining previously endemic villages and to initiate a supplemental MDA programme to include any village found to have prevalence rates of 1% or more. This remains an essential step prior to certifying Egypt as country free of LF. Therefore, data from the present study are of great importance to MOHP decision makers for developing a supplemental programme expansion, with a realistic budget estimate and for raising funds essential for its implementation.

## Conclusion

The present study estimated the overall Total and Government costs of MDA in LF-endemic villages in the Nile Delta and the governorates of Giza and Asiut in Upper Egypt. Approximately 1,795,553 individuals, living in 161 villages in 7 governorates, were treated by MDA in the year 2000 with a coverage rate of 86.0%. The overall Total and Government costs for MDA that year were US $3,181,000 and US $2,412,000, respectively. Average Total and Government costs per treated subject and person at risk in 2000 were US $1.77 and US $1.34, respectively. In 2001, while the number of persons treated increased (29%) and the coverage rate increased to 88.0%, the Total and Government costs decreased to US $3,109,000 and US $2,331,000, respectively. Consequently, the average Total and Government costs per treated subject and per person at risk decreased to US $1.34 and US $1.00, respectively.

National agencies of Egypt contributed 75.8% of the Total costs, a fact highlighting the commitment of the Egyptian government toward the LF elimination programme but not minimizing the significance of the contributions of international bodies, such as WHO, EMRO, GSK and the Arab Fund for Economic and Social Development.

The results of this study set the stage for the MOHP to initiate a supplemental MDA programme expansion, estimate a realistic budget and raise essential funds for its implementation. The objectives of the supplemental programme are to map villages with a previous history of LF, not included in the current programme, and ensure coverage of eligible villages with needed MDA rounds. This action is necessary prior to certifying Egypt as a country free of LF.

## Competing interests

The author(s) declare that they have no competing interests.

## Authors' contributions

RMRR participated in the study design and its coordination, data collection and analysis, and drafted the manuscript. ASG designed and modified the generic protocol to suit the Egyptian programme and participated in writing the manuscript. HAK shared in the study design, data collection and reviewed the manuscript. All authors read and approved the final manuscript.
